# Exploring College Students’ AI Dependence in Human–AI Collaborative Creation: Development and Validation of the CC-PACE Model

**DOI:** 10.3390/jintelligence14080155

**Published:** 2026-08-01

**Authors:** Xuping Xiong, Fuhai An

**Affiliations:** 1 Jing Hengyi School of Education, Hangzhou Normal University, Hangzhou 311121, China; xxp1996@126.com; 2 Chinese Education Modernization Research Institute, Hangzhou Normal University, Hangzhou 311121, China

**Keywords:** AI dependence, creativity, the CC-PACE model, the 5A’s framework, the I-PACE model

## Abstract

The development of Artificial Intelligence (AI) poses significant challenges to creativity research and practice. Managing the degree of AI dependence during the creative process is therefore critical to fostering the healthy development of human creativity. To address this concern, this study proposes the CC-PACE model by integrating the 5A’s framework with the I-PACE model, and examines factors associated with AI dependence during the creative process. A cross-sectional survey was conducted with 813 college students in China, and the data were analyzed using partial least squares structural equation modeling (PLS-SEM). The results showed that (1) both creative personal identity and creative self-efficacy were positively associated with closeness with the audience and the perception of AI as a creative agent. (2) Both closeness with the audience and the perception of AI as a creative agent were positively associated with time spent using AI, usage preference, and perceived difficulty in avoiding AI use. (3) Time spent and usage preference were positively associated with students’ creative self-assessment, whereas perceived difficulty in avoiding AI use was not significantly associated with creative self-assessment. This study provides a viable analytical framework for understanding AI dependence in creative contexts. The findings further suggest that teachers should help students develop an appropriate understanding of AI’s role, strengthen students’ creative agency, and emphasize process-oriented assessment in creative activities.

## 1. Introduction

**Figure 1:**
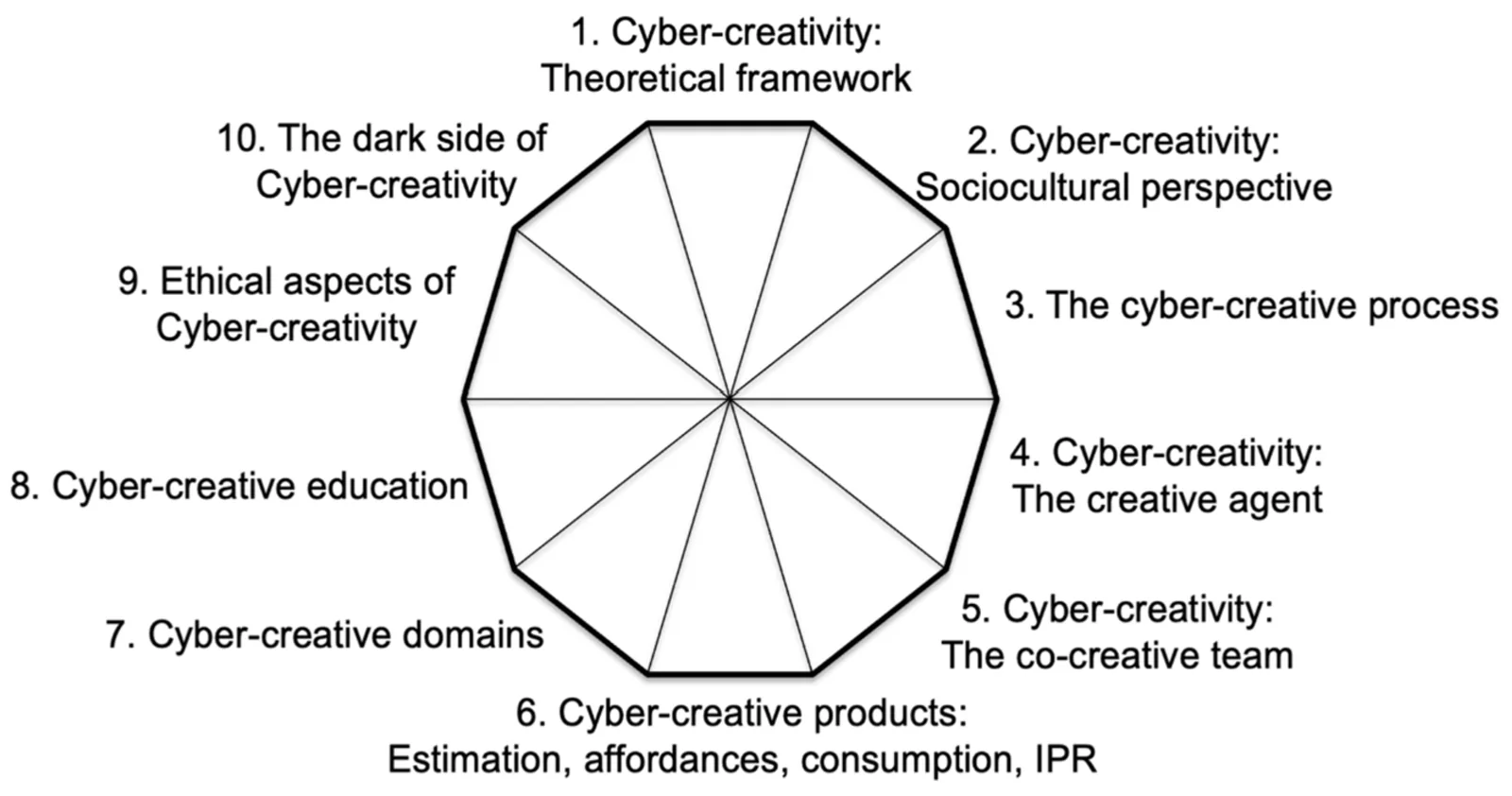
The “Decalogue” of challenges for research in cyber-creativity (Corazza et al., 2025).

Creativity has been identified as a key driver of cultural progress (Thornhill-Miller et al., 2023). The expanding potential of Artificial Intelligence (AI) in creative work and its wide range of applications highlights a critical question: how can AI be used to promote creativity while mitigating risk? To foster synergy between human creativity and AI, 30 expert members of the AI Task Force of the International Society for the Study of Creativity and Innovation (ISSCI), including Giovanni Emanuele Corazza, Sergio Agnoli, and Ana Jorge Artigau, collectively proposed the “Decalogue” of research challenges (Corazza et al., 2025) (Figure 1).

The “Decalogue” provides a comprehensive conceptual framework for guiding creativity research in the context of AI. The framework consists of ten dimensions and 60 specific research questions. Drawing on this framework, we identified a central theme that cuts across several dimensions, namely creators’ AI dependence during the creative process. Specifically, the framework suggests that creators’ personal characteristics, affective responses, and cognitive processes may be associated with AI dependence in creative activities (Dimension 4). AI may play different roles across stages of the creative process (Dimension 3), while individuals’ understanding of AI’s role (Dimension 5) may shape their engagement with AI and the development of the creative process. And the use of AI may shape how creative products are evaluated (Dimension 6). In educational contexts, the potential negative consequences of AI use have received growing attention in creativity education (Dimension 8). Clarifying these interconnected mechanisms can therefore provide a more robust theoretical basis and practical implications for creativity research and creativity education in the age of AI. Accordingly, the present study addresses the following three research questions.

RQ1: How are personal characteristics associated with affective and cognitive responses during the creative process?

RQ2: How are affective and cognitive responses associated with dependence on AI during creative processes?

RQ3: How is AI dependence during creative processes associated with creative product evaluation?

This study aims to examine the interrelated factors associated with students’ AI dependence in creative processes. Based on the Interaction of Person–Affect–Cognition–Execution (I-PACE) model and the 5A’s framework of creativity, this study proposed the C-Creativity of Person–Affect–Cognition–Execution (CC-PACE) model to explore the potential mechanisms underlying this dependence. This model provides a reference for future research on AI dependence in creative processes and offers pedagogical guidance to teachers to support students in the appropriate use of AI.

### 1.1. The I-PACE Model

Theoretical models that explain the development and maintenance of Internet-use disorders provide an important basis for understanding technology-related dependence. Previous studies have proposed several explanatory models from different theoretical perspectives. For instance, Brand et al. (2014) developed a model of specific Internet-use disorders from a neuropsychological perspective, whereas Dong and Potenza (2014) proposed a cognitive–behavioral model of Internet gaming disorder. These models have provided important theoretical foundations for understanding Internet-use disorders. However, because Internet-use disorders involve a wide range of interacting factors, models grounded in a single theoretical perspective tend to capture only part of this complexity. In this context, Brand et al. (2016) proposed the Interaction of Person–Affect–Cognition–Execution (I-PACE) model, which provides a more integrative theoretical framework. This model was designed to explain the development and maintenance of Internet-use disorders. As a process model, it integrates theoretical and empirical findings from multiple domains and emphasizes the interactions among the following components: predisposing variables, affective and cognitive responses to internal or external stimuli, executive and inhibitory control, decision-making behaviors resulting in the use of specific Internet applications or platforms, and the consequences associated with such use.

The first core component of the I-PACE model is predisposing variables, the “P” com-ponent, which represent core personal characteristics. These characteristics have been identified as key risk factors for the dependence. For example, genetic factors (Deryakulu & Ursavaş, 2014) and personality traits (Ebeling-Witte et al., 2007; C. W. Wang et al., 2015) have been found to be significantly associated with Internet use, and adverse childhood experiences could increase the risk of Internet-use disorders (Hsieh et al., 2016). The second core component of the model, denoted by “A”, represents affective responses to external or internal stimuli. These responses can give rise to emotional states, which may influence decision-making processes. For instance, people may exhibit different decision-making responses under different levels of stress (Starcke & Brand, 2012). The third component, denoted by “C”, represents cognitive responses to internal or external stimuli. For example, Internet-use-related metacognitions and use expectancies have been found to be associated with Internet-use behavior (Casale et al., 2016; Wegmann et al., 2015). Components “A” and “C” have been identified as significant factors contributing to the development of dependence. “E”, the fourth core component of the model, pertains to the execution stage. For example, reduced inhibitory control may contribute to the development of addiction (Goldstein & Volkow, 2011). Impairments in executive functions, reflected in indicators such as reaction time and accuracy, have also been found to be associated with Internet addiction (Dong et al., 2011). The fifth component is the consequences of using the Internet. Internet-use behaviors may result in positive experiences or satisfaction, which reinforce affective or cognitive responses and create a reinforcing cycle (Brand et al., 2016).

In summary, the I-PACE model provides a comprehensive theoretical framework for examining Internet-use disorders. Each component incorporates key factors that have been theoretically proposed or empirically validated. The model can be applied broadly across studies of technology-related dependence, such as gaming addiction (Young & Brand, 2017), addictive short-video use (Jiang et al., 2025), and problematic Internet use (Stanciu & Calugar, 2022). In the context of AI use, S. Zhang et al. (2024) explored the associations between academic self-efficacy, academic stress, performance expectations, and AI dependence from the perspective of the “P” component of the model. In a subsequent study, Ye et al. (2025) investigated the relationships among avoidance learning motivation, positive experiences, and inert thinking regarding AI dependence, corresponding to the four components from “P” to “E”. Researchers could select specific factors and integrate them into the relevant components to analyze their effects (Brand et al., 2016). Although previous research has linked AI dependence with various aspects of student development, studies focusing specifically on creativity-related factors remain limited and require further empirical investigation.

### 1.2. The 5A’s Framework

A growing body of research has examined human–AI collaborative creation, introducing several related concepts. For example, “Co-creativity” refers to creative collaboration in which humans and AI systems act as partners (McGuire et al., 2024). “Cyber-creativity” also describes collaboration on creative tasks between one or more human agents and one or more AI machines (Corazza et al., 2025). Despite their terminological differences, both concepts emphasize collaborative creative processes in which humans and AI engage jointly in diverse forms of creative activity. Given this conceptual overlap, this study uses the term “C-creativity” as an umbrella term to encompass these related concepts.

The 5A’s framework is one of the most widely used frameworks in the field of creativity (Barón-Birchenall et al., 2025; Corazza et al., 2025; Thornhill-Miller et al., 2023). Glăveanu (2013) proposed this framework by expanding the 4P’s model (Rhodes, 1961), which consists of four elements: Person, Process, Product, and Press. The 5A’s framework extends this model into a culturally and socially grounded version that includes five interrelated elements: Actor, Action, Artifact, Audience, and Affordances. In the context of C-creativity, AI is also connected to each of these elements.

In the 5A’s framework, the actor refers to the individual who engages in creative activity. Glăveanu (2013) emphasizes that the actor cannot be understood independently of its context. Although creative activity is shaped by individual characteristics, it is also influenced by social relations and cultural traditions. In contemporary creative practices, AI constitutes an important technological context that may shape individuals’ creative behaviors and psychological processes. For example, recent research suggests that the use of AI is associated with variations in individuals’ creative self-efficacy (Sabrina, 2026). The second element is action. It encompasses both psychological and material dimensions. It is internal and external, goal-directed, structured, and symbolically meaningful. AI can support the creative process in various ways. For example, visual AI can provide ideas and resources during creation (Rafner et al., 2023), while relay-style human–AI creation expands the possible modes of the creative process (McGuire et al., 2024). The third element, artifact, possesses a dual nature, encompassing both material and conceptual or ideal dimensions. A growing body of research has demonstrated the influence of AI on creative outcomes. For example, AI has been shown to support individuals in generating more creative ideas for problem solving (Bai, 2026) and in producing higher-quality creative works (Doshi & Hauser, 2024). However, some studies have also found that the quality of individuals’ creative works may decline after AI assistance is removed (Wong & Qiu, 2026). These findings suggest that AI may exert multiple influences on this element. Moreover, the artifact needs to be evaluated within the context of its formation process, the socio-cultural environment, and the creator, rather than as an isolated “object”. This relational perspective is particularly relevant to C-creativity, where AI use may introduce biases into creators’ self-evaluations of their creative works (Urban et al., 2024). Audience is the fourth element in the 5A’s framework. Each actor’s audience may vary widely, including family members, competitors, coworkers, and even the broader public. Creation is inherently dialogic (Grossen, 2008), and the audience plays an important role as an evaluator and a co-constructor of meaning. During the creative process, actors often need to anticipate and interpret their work from the audience’s perspective. Recent studies have shown that, as AI becomes increasingly widespread, audiences place strong emphasis on human supervision over C-creativity (Zier & Diakopoulos, 2026). Different audience groups also vary in their acceptance of AI technology. For instance, novelty-seeking individuals are more likely to accept AI-generated creative products (Cao & Wang, 2025). Thus, in C-creativity, the audience’s acceptance of AI has also become a factor that actors need to take into consideration. The final element is affordances. The material, instrumental, functional, communicative, and symbolic characteristics of objects shape different aspects of action and guide the actor’s engagement. Individuals first perceive the affordances in their environment, and as their competencies develop, they become increasingly proficient in using them. They may then actively modify or reshape them, leading to transformative or innovative outcomes. This process also applies to actor’s use of AI. AI has been shown to be useful across multiple domains, including painting (Sun & Qin, 2024), writing (McGuire et al., 2024), and data analysis (X. M. Wang et al., 2023). It is also regarded as having various potential functions, such as coordination, creation, refinement, and execution (Siemon, 2022). Sidra and Mason (2026) showed that people’s productivity is influenced by their ability to recognize AI applications across domains, understand how AI can be used, and identify the functions it can perform. This suggests that people’s perceptions of AI “affordances” shape their use of AI, thereby contributing to different creative and productivity-related outcomes.

In conclusion, the 5A’s framework for creativity emphasizes the connection and unity among the five elements, as well as the socio-environmental factors underlying them. As technology advances, AI has begun to demonstrate levels of creativity that approach or even match those of professional creators. This development suggests that AI will likely affect the elements of the 5A’s framework. Although AI is not yet fully autonomous, many scholars believe that its current limitations have significant potential for change (Corazza et al., 2025). Consequently, researchers must recognize AI as a critical and urgent challenge for creativity research. While exploring AI’s potential to enhance creativity, they also need to remain vigilant about its potential risks. The double-edged sword nature of AI has thus emerged as a critical concern in contemporary creativity research (Corazza et al., 2025).

### 1.3. The CC-PACE Model and Research Hypothese

AI interventions could affect individuals’ creative ideas, skills (Bakker et al., 2022), and processes (Berretta et al., 2023). Preventing AI dependency in C-creativity has emerged as a central research theme (Corazza et al., 2025). The 5A’s framework captures key creativity-related elements, but it does not specify the relationships among these elements. Glăveanu (2013), who proposed the 5A’s framework, also regarded this as an important issue for future research. The I-PACE model proposes dependence-related components and their potential relationships. However, the specific factors involved may vary across different dependence. The components in the I-PACE are relatively broad rather than specific, and they are not specifically tailored to the creativity domain. Therefore, when examining AI dependence in C-creativity, the five components of the I-PACE model need to be operationalized as concrete creativity-related factors, and the 5A’s framework offers a theoretically grounded set of such factors. Therefore, we integrated them into the following five constructs: Actor-Person, Audience-Affect, Affordances-Cognition, Action-Execution, and Artifact-Consequence. The theoretical relationships among these five constructs were established based on the logic underlying the I-PACE model’s connections among Person, Affect, Cognition, Execution, and Consequence. The resulting model was named the C-Creativity-PACE (CC-PACE) model. The study selected five factors to validate the five proposed constructs of the CC-PACE model (Table 1): self-concept of creativity, closeness with the audience, AI roles, AI dependence in C-creation, and creative assessment.

**Figure 2:**
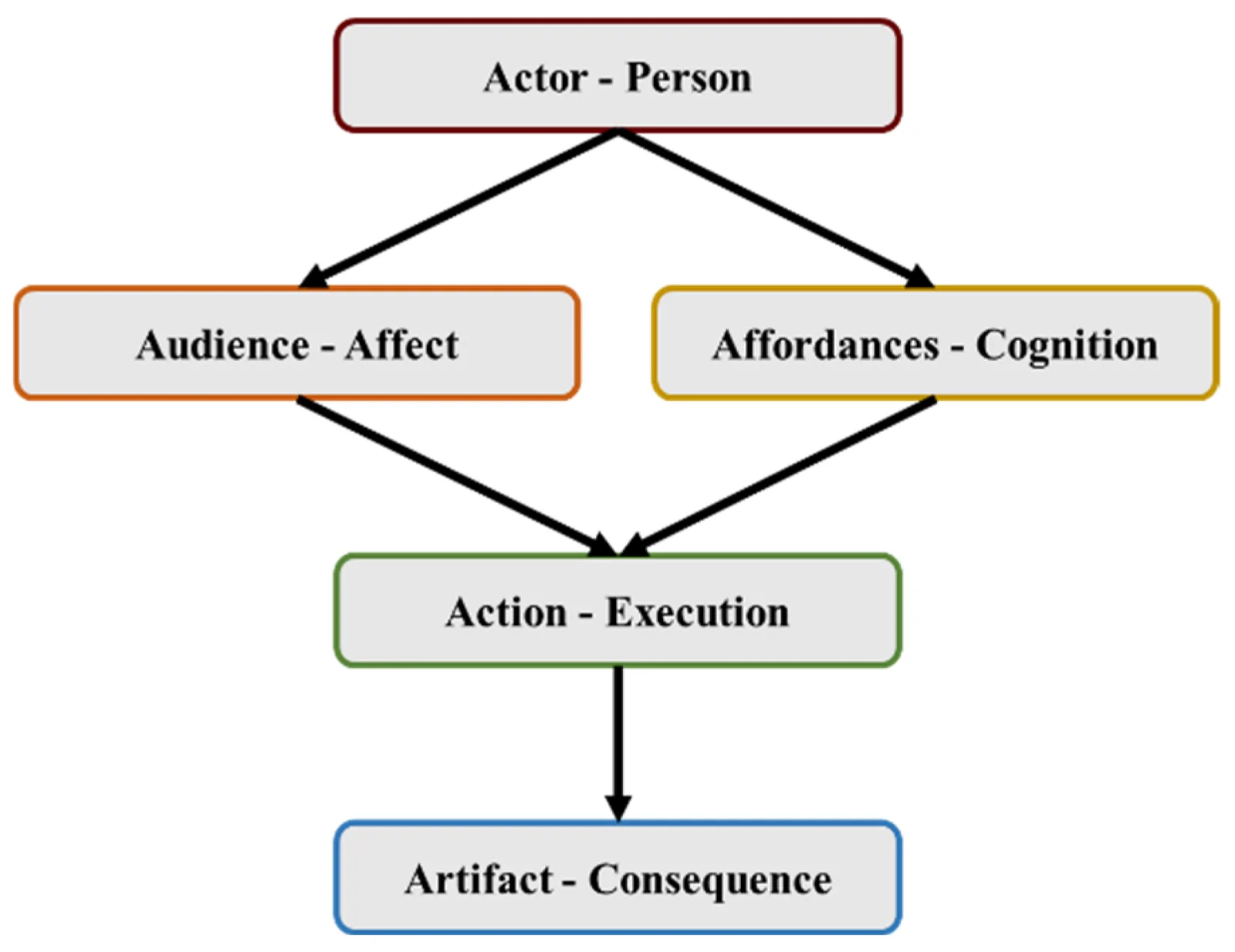
The CC-PACE model.

Based on the logic provided by the I-PACE model, we mapped the potential relationships among the five constructs of CC-PACE, as shown in Figure 2. Actor-Person, which reflects individual characteristics related to creativity, serves as the starting point, and these personal factors may influence Audience-Affect and Affordances-Cognition. These affective and cognitive responses may further shape specific creative behaviors, represented by the Action-Execution construct. Creative behaviors then influence the final creative outcomes, represented by Artifact-Consequence. Although the I-PACE model implies potential reciprocal and reinforcing processes over time, the present study focuses on the primary relationships among the proposed constructs in order to provide a parsimonious and testable framework.

#### 1.3.1. Actor-Person

The actor in the 5A’s framework and the person in I-PACE model both refer to individual-level factors related to the creator. Therefore, when examining AI dependence in C-creativity, they can be integrated into the combined construct of Actor-Person, which encompasses factors associated with individual characteristics.

Creative self-concept is an important construct in creativity research and comprises creative personal identity (CPI) and creative self-efficacy (CSE) (Karwowski et al., 2013) Accordingly, CPI and CSE were selected as indicators of the Actor-Person construct in the CC-PACE model. CPI is a self-perception that creativity constitutes a central and defining aspect of one’s identity. Individuals with a high CPI view creativity as integral to their self-definition (Karwowski et al., 2013). CSE is defined as one’s confidence in their ability to address tasks that require creative thinking and creative performance. It has been associated with higher levels of creative achievement.

According to the CC-PACE model, Actor-Person, which includes CPI and CSE, may influence variables within the Audience-Affect and Affordances-Cognition constructs. Thus, the following hypotheses are proposed:

**H1.**  *CPI is positively and significantly associated with CA.*

**H2.**  *CPI is positively and significantly associated with PAICA.*

**H3.**  *CSE is positively and significantly associated with CA.*

**H4.**  *CSE is positively and significantly associated with PAICA.*

#### 1.3.2. Audience-Affect

The 5A’s framework identifies the audience as an important element of creativity. Creators often need to consider audience perspectives during the creative process (Grossen, 2008). Compared with conventional creative works, works produced through C-creativity may prompt audiences to evaluate the relative contributions of AI and humans to their production (Zier & Diakopoulos, 2026), and audiences may differ in their acceptance of AI involvement in creative works (Cao & Wang, 2025). Accordingly, when engaging in C-creativity, creators may experience varying emotional responses when anticipating how different audiences will perceive AI involvement in their work. This notion is related to the affective-response component of the I-PACE model. Therefore, we integrated the audience element from the 5A’s framework with the affective-response component of the I-PACE model to form the Audience-Affect construct, which captures these audience-related emotional responses.

During the creative process, these emotional responses are primarily associated with creators’ expectations of how the audience will perceive them. Individuals tend to expect closer audiences to communicate more positively, understand them better, and provide greater support (Y. Zhang et al., 2022). Accordingly, creators may experience varying emotional responses when anticipating evaluations from audiences with different levels of closeness. In this study, we therefore use closeness with the audience (CA) as an indicator of the Audience-Affect construct in the CC-PACE model. CA refers to creators’ perceived relational proximity to the potential audience or evaluators of their creative work.

According to the CC-PACE model, Audience-Affect may influence variables within the Action-Execution construct constructs. Thus, the following hypotheses are proposed:

**H5.**  *CA is positively and significantly associated with TS.*

**H6.**  *CA is positively and significantly associated with UP.*

**H7.**  *CA is positively and significantly associated with PDA.*

#### 1.3.3. Affordances-Cognition

In the context of C-creativity, the affordances element in the 5A’s framework refers to the action possibilities offered by AI, which creators gradually come to understand through use. This understanding reflects creators’ cognitive responses to the tool and corresponds to the cognitive response component of the I-PACE model. Given this conceptual similarity, we integrated the affordances element of the 5A’s framework with the cognitive response component of the I-PACE model to form the Affordances-Cognition construct, which captures creators’ cognitive understanding of AI affordances.

This cognitive understanding can be operationalized through creators’ perceptions of AI’s role in C-creativity. In this study, we focus on whether creators perceive AI as capable of functioning as a creative agent. Accordingly, we use the perception of AI as a creative agent (PAICA) as an indicator of the Affordances-Cognition construct in the CC-PACE model. The PAICA signifies the degree to which actors agree that AI can serve as a creator in C-creation and that its generative capabilities can support the effective execution of C-creative activities. PAICA reflects actors’ cognizance of the affordances associated with using AI as a creative tool.

According to the CC-PACE model, Affordances-Cognition may influence variables within the Action-Execution construct constructs. Thus, the following hypotheses are proposed:

**H8.**  *PAICA is positively and significantly associated with TS.*

**H9.**  *PAICA is positively and significantly associated with UP.*

**H10.**  *PAICA is positively and significantly associated with PDA.*

#### 1.3.4. Action-Execution

The action element of the 5A framework captures the creative process. In the context of C-creativity, creators use AI to support their creative activities during this process. Accordingly, this process corresponds to the execution component of the I-PACE model, which refers to behaviors in using a particular technology. Given this conceptual correspondence, we integrated the action element of the 5A’s framework with the execution component of the I-PACE model to form the Action-Execution construct. This construct represents the process that people use AI to create and provides a direct indication of creators’ dependence on AI during the creative process.

In the context of C-creativity, AI dependence refers to the extent to which creators rely on AI during C-creative processes. According to Andreassen et al.’s (2012) investigation of technological dependence, it can be measured in terms of time spent (TS), usage preference (UP), and perceived difficulty in avoiding AI use (PDA). These factors occur during C-creative processes and reflect actors’ engagement with the technology during execution. Therefore, we selected these three variables as indicators of the Action-Execution construct in CC-PACE.

According to the CC-PACE model, Affordances-Cognition may influence variables within the Artifact-Consequence construct. Thus, the following hypotheses are proposed:

**H11.**  *TS is positively and significantly associated with CSA.*

**H12.**  *UP is positively and significantly associated with CSA.*

**H13.**  *PDA is positively and significantly associated with CSA.*

#### 1.3.5. Artifact-Consequence

The artifact element of the 5A’s framework represents the final creative product. It also corresponds to the outcome generated through the use of technology in C-creativity and aligns with the consequence component of the I-PACE model. Therefore, we integrated them to form the Artifact-Consequence construct, which represents the outcomes of C-creativity.

To assess these outcomes, we focused on creators’ perceived quality of their creative products, as self-assessment provides a direct account of how creators evaluate the quality and value of their own creative work. Therefore, we use creative self-assessment (CSA) as an indicator of the Artifact-Consequence construct in the CC-PACE model.

**Figure 3:**
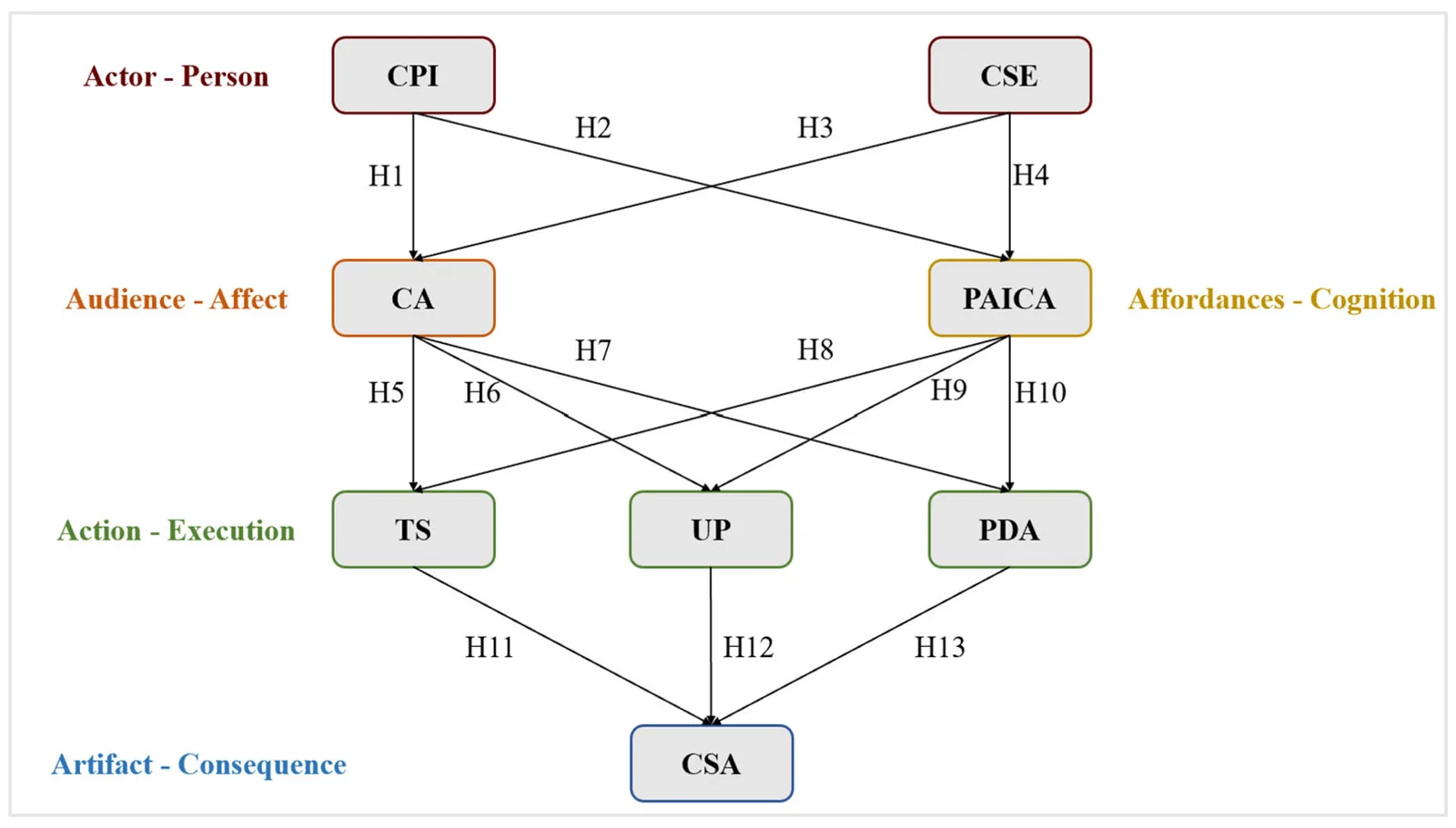
Research hypothesis.

Figure 3 shows the hypothesized model of this study.

## 2. Materials and Methods

### 2.1. Participants

The survey used convenience sampling, and questionnaires were distributed to students at a university in eastern China via Wenjuanxing, an online survey platform. As the sample was drawn from a single university using convenience sampling, the findings should be interpreted with caution in terms of their generalizability. The electronic questionnaire included an informed consent statement, and participants were informed about the study’s purpose and their role. All participants had experience with C-creation. Their age and gender characteristics are presented in Table 2. The data collection period lasted from 5 January to 15 January 2026.

A total of 813 valid responses were retained for the final analysis, exceeding the minimum required sample size. G*Power (version 3.1.9.7) was used to estimate the minimum sample size (Faul et al., 2009, 2007), and the results indicated a minimum of 77 participants. The sample size also satisfied the 10-times rule commonly applied in PLS-SEM, which requires at least ten times the maximum number of structural paths directed at any endogenous construct (Baudier et al., 2025).

### 2.2. Measures

The main body of the questionnaire consisted of 40 questions, including 39 mandatory single-choice items and one optional short-answer question. The selection and adaptation of items were guided by three considerations. First, each item was required to be theoretically aligned with the constructs examined in this study. Second, items were selected or adapted from established scales to maintain conceptual consistency with prior research. Third, items that were redundant or irrelevant to the study context were removed to ensure that the questionnaire remained concise and suitable for student participants. The adapted questionnaire was then reviewed by experts to assess the clarity, contextual relevance, and theoretical coherence of the selected items.

All questions were presented in Chinese. All items were presented in Chinese. The English items were first translated into Chinese by a bilingual researcher familiar with the research topic. The translated items were then reviewed by another bilingual researcher to ensure linguistic accuracy, conceptual consistency with the original items, and contextual suitability for student participants. Before questionnaire distribution, the research team reviewed the Chinese version and revised any wording that was unclear or potentially ambiguous.

**Figure 4:**
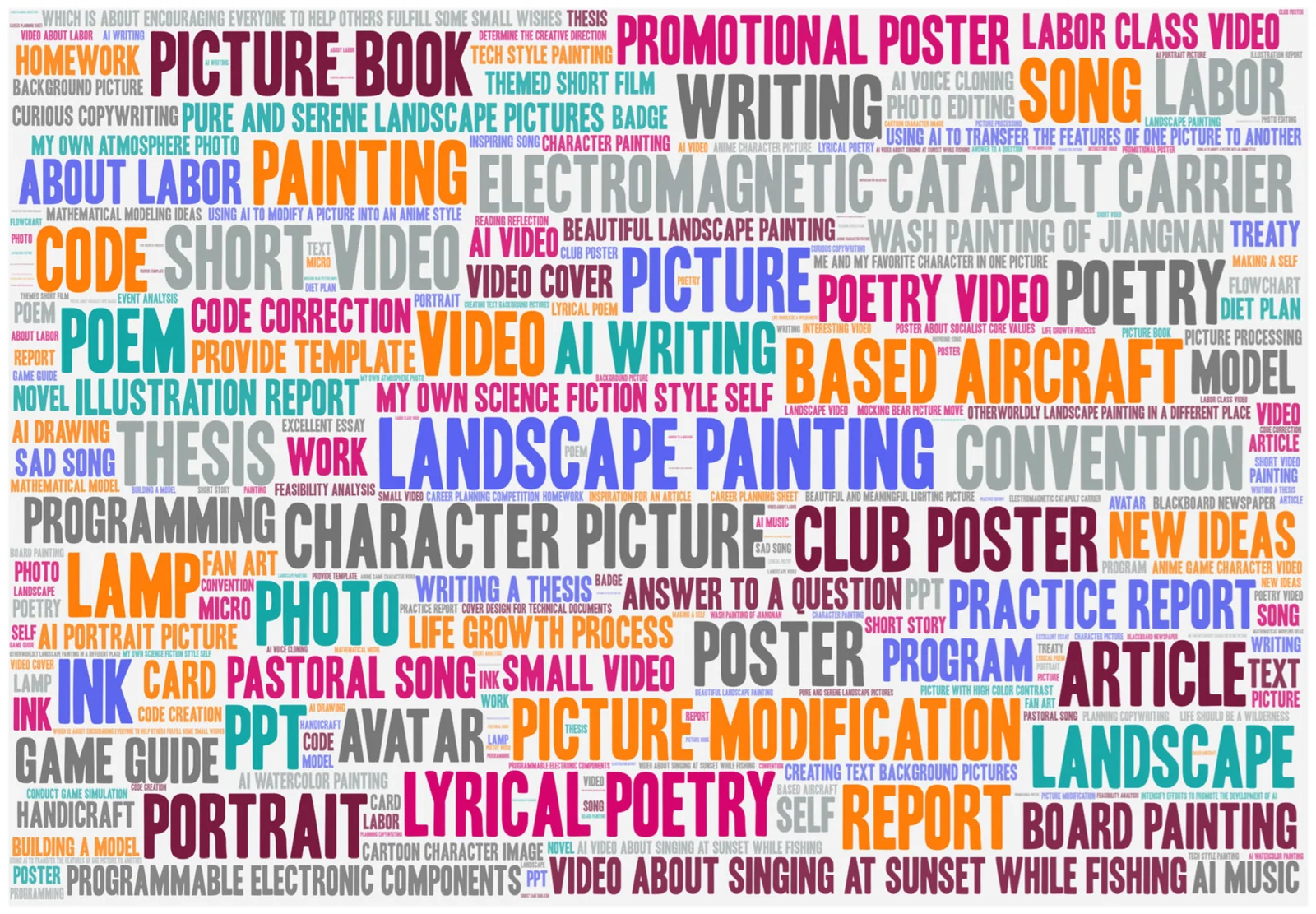
The types of C-creative work reported by the students.

The single-choice items used a five-point Likert scale (1 = Strongly disagree, 2 = Disagree, 3 = Neutral, 4 = Agree, 5 = Strongly agree). The first section of the questionnaire examined students’ CPI, CSE, and PAICA. The second section then asked students to recall a recent or particularly memorable instance of creative work completed through collaboration with AI. At this stage, an optional open-ended question was included, allowing students to provide information about the specific C-creative work if they were willing to do so. Figure 4 illustrates the types of C-creative works students reported. The subsequent items assessed CA, TS, UP, PDA, and CSA in relation to the C-creative work that students recalled.

#### 2.2.1. The Short Scale of Creative Self

The self-concept of creativity was measured using the Short Scale of Creative Self (SSCS) developed by Karwowski et al. (2013), which comprises two components: the CPI and the CSE. The CPI section includes five items, for example, “I think I am a creative person.” A higher CPI score indicates a stronger identification with one’s creative identity. The CSE section includes six items, for example, “Many times I have proved that I can cope with difficult situations.” A higher CSE score reflects greater confidence in one’s ability to engage in creative activities.

#### 2.2.2. The Modified Miller’s Social Intimacy Scale

The CA was measured using a modified version of Miller’s Social Intimacy Scale (Y. Zhang et al., 2022). This section included three items, for example, “I think these people could understand me.” A higher score indicates that the actor perceived greater closeness to the actual or imagined audience.

#### 2.2.3. AI-Based Team Roles

The survey items used to measure PAICA were derived from Siemon’s (2022) framework of four AI-based team roles. Specifically, six descriptions of AI as a creator in Siemon’s framework were adapted as survey items to examine students’ perceptions of AI as a creator. The adapted items are presented in Table 3. Expert review provided evidence for the content validity of these items, indicating that they preserved the conceptual meaning of the original descriptions and appropriately captured students’ PAICA. Higher scores indicate stronger students’ perceptions of AI as capable of functioning as a creator.

#### 2.2.4. The Addiction Scale

AI dependence was assessed using an adapted version of the addiction scale developed by Andreassen et al. (2012). The original scale evaluates addictive tendencies by measuring key behavioral symptoms, including Salience, Tolerance, and Relapse. For the present study, three items were selected because they most closely reflected students’ AI-dependent behaviors during C-creation processes, including TS, UP, and PDA. To better align the measure with the creativity-related context of this study, these items were rephrased to capture students’ use of AI in C-creativity.

This adaptation was guided by the Creative Problem Solving (CPS) model; the creative process consists of four components: understanding the challenge, generating ideas, preparing for action, and planning the approach. TS, UP, and PDA were measured at each stage to provide a comprehensive assessment of students’ AI dependence. The adapted scale is presented in Table 4. This adaptation aimed to enhance its relevance to C-creation processes. Items were selected and modified based on their theoretical alignment with the target constructs and their consistency with the CPS framework.

The adapted questionnaire was reviewed by experts in creativity research. The experts judged the adapted items to be relevant to the research context and aligned with the intended constructs. They also endorsed the expansion of each selected item from the original addiction scale into four CPS-based sub-items. The expert review provided preliminary evidence for the content validity of the adapted questionnaire.

#### 2.2.5. Creative Self-Assessment

The creativity of students’ works was evaluated using self-assessment. There were two reasons for selecting self-assessment rather than expert evaluation. First, it reflects students’ perceptions of their own creative performance in C-creation contexts, thereby allowing the study to examine the impact of AI use on students’ subjective feelings. Second, self-assessment is more feasible for large-scale survey.

The CSA section asked students to evaluate their work on fluency, flexibility, and originality. These dimensions are commonly used in assessing artifacts (Amabile, 1982). This section consists of three items, each corresponding to one of these dimensions, such as the originality item: “Compared with other people’s work, my piece is more innovative and unique in its ideas, mode of expression, or form of presentation.” Higher scores indicate that students perceived their work as more creative and of higher quality.

### 2.3. Statistical Tools

This study aimed to investigate the relationship among CPI, CSE, CA, PAICA, TS, UP, PDA, and CSA. First, descriptive statistical analyses were conducted using SPSS 26.0. Second, the hypothesized research model was tested using SmartPLS 4, based on partial least squares structural equation modeling (PLS-SEM). PLS-SEM is widely regarded as an important multivariate analysis approach, as it is capable of handling highly complex models and offers advantages for validating theoretical relationships and assessing model fit (Magno et al., 2022; Zyphur et al., 2023).

## 3. Results

### 3.1. Descriptive Analysis

Descriptive statistics indicate that the measured constructs demonstrated reasonable mean scores (Table 5). PDA showed the highest mean (M = 2.603, SD = 0.842), whereas CPI had the lowest (M = 1.865, SD = 0.660).

### 3.2. Measurement Model Assessment

The reliability and validity of the measurement model were evaluated through four procedures. First, reliability was assessed through Cronbach’s alpha and composite reliability (CR). Second, convergent validity was evaluated using average variance extracted (AVE) and indicator loadings. Third, discriminant validity was tested based on the Fornell-Larcker criterion and the Heterotrait–Monotrait ratio of correlations (HTMT). Finally, variance inflation factor (VIF) values were examined to assess potential multicollinearity issues.

For reliability, Cronbach’s alpha and CR values greater than 0.70 indicate adequate internal consistency. As shown in Table 6, all constructs met these recommended thresholds, suggesting that the measurement model demonstrated satisfactory reliability.

Convergent validity was assessed by examining whether AVE values exceeded 0.50. All latent variables in Table 6 had AVEs above the recommended level, indicating adequate convergent validity for the constructs. Additionally, indicator loadings were examined. All items had loadings above 0.70, further confirming that the indicators reliably captured their respective constructs.

Discriminant validity was first examined using the Fornell-Larcker criterion. According to this criterion, the square root of AVE for each construct should exceed its correlations with other constructs. As reported in Table 7, the diagonal values were higher than the corresponding off-diagonal inter-construct correlations, indicating adequate discriminant validity across the eight constructs. Discriminant validity was further assessed using the HTMT criterion, which is widely recommended for variance-based PLS-SEM (Hair et al., 2019). A commonly accepted threshold for HTMT is 0.85, and Table 8 consequently indicates satisfactory discriminant validity.

Finally, VIF values were examined to diagnose multicollinearity. VIF values below 5 indicate the absence of serious collinearity concerns. The results showed that all VIF values ranged from 2.214 to 4.456, suggesting that multicollinearity was not a concern in this study.

### 3.3. Structural Model Assessment

In PLS-SEM analyses, it is important to evaluate both explanatory power and predictive accuracy, rather than emphasizing overall model fit (Hair et al., 2019; Magno et al., 2022). Therefore, the coefficient of determination (R^2^), the path coefficients (*β*), the effect size (f^2^), and predictive relevance (Q^2^) were evaluated to assess the model’s explanatory and predictive power. R^2^ indicates the proportion of variance in each endogenous construct explained by its predictors (0–1), with higher values indicating greater explanatory power. Path coefficients typically range from −1 to +1, where larger absolute values denote stronger effects. To assess statistical significance, a bootstrap procedure with 5000 samples was performed. The effect sizes (f^2^) statistic evaluates the unique contribution of each predictor to an endogenous construct, with thresholds of 0.02 (small), 0.15 (medium), and 0.35 (large). Below are the results for the six endogenous constructs.

Research question 1 was addressed through hypotheses 1–4, all of which were supported (Table 9). Specifically, CPI was positively associated with CA (*β* = 0.153, *t* = 2.845, *p* = 0.004) and PAICA (*β* = 0.234, *t* = 3.925, *p* < 0.001). CSE was also positively associated with CA (*β* = 0.387, *t* = 7.084, *p* < 0.001) and PAICA (*β* = 0.403, *t* = 6.761, *p* < 0.001).

Research question 2 was addressed through hypotheses 5–10, all of which were supported (Table 9). Specifically, CA was positively associated with TS (*β* = 0.333, *t* = 7.687, *p* < 0.001), UP (*β* = 0.320, *t* = 7.004, *p* < 0.001), and PDA (*β* = 0.281, *t* = 5.841, *p* < 0.001). PAICA was also positively associated with TS (*β* = 0.374, *t* = 8.867, *p* < 0.001), UP (*β* = 0.347, *t* = 7.894, *p* < 0.001), and PDA (*β* = 0.216, *t* = 5.101, *p* < 0.001).

Research question 3 was addressed through hypotheses 11–13. H11 and H12 were supported, whereas H13 was not supported (Table 9). Specifically, TS (*β* = 0.374, *t* = 7.324, *p* < 0.001) and UP (*β* = 0.251, *t* = 4.324, *p* < 0.001) were positively associated with CSA. In contrast, PDA was not significantly associated with CSA (*β* = 0.079, *t* = 1.930, *p* = 0.054).

The bootstrapped 95% confidence intervals for the supported path coefficients did not include zero, and all significant relationships were positive.

Among these supported paths, the f^2^ were further examined to assess their practical significance. The association between CPI and CA (f^2^ = 0.014 < 0.02) fell below the threshold for a small effect, indicating limited practical significance despite being statistically significant. In contrast, the association between PAICA and TS had a medium effect size (f^2^ = 0.168 > 0.15), suggesting that PAICA made a practically meaningful contribution to explaining students’ actual time spent using AI. The remaining supported paths had small effect sizes (0.02 < f^2^ < 0.15), indicating that these predictors made statistically significant but modest contributions to their respective endogenous constructs.

**Figure 5:**
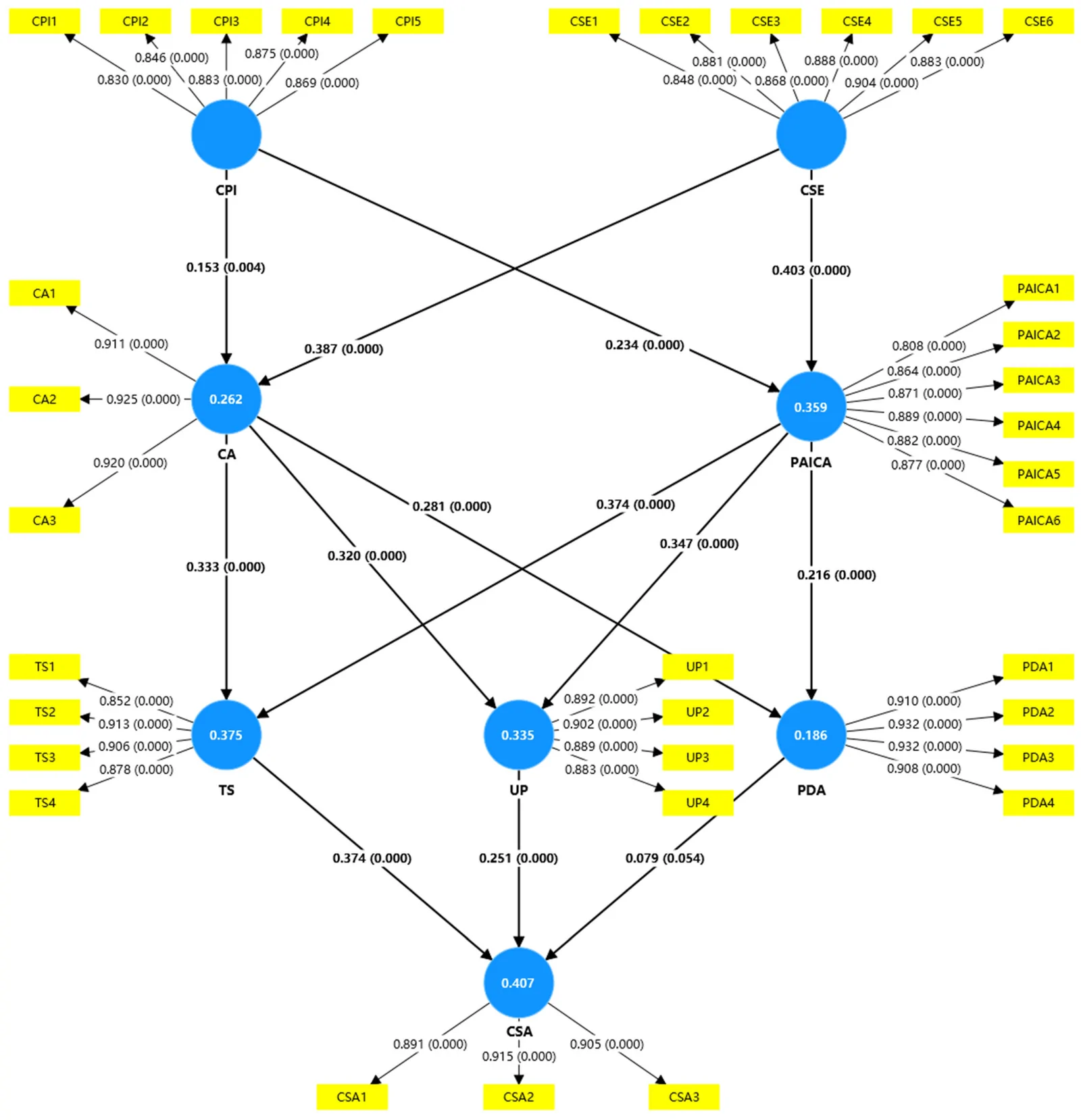
Research model with results.

Finally, Q^2^, obtained through the blindfolding procedure, assesses the predictive relevance of the model, indicating its ability to predict omitted data beyond the observed sample. All Q^2^ values in Table 10 were greater than zero, demonstrating predictive relevance for the endogenous constructs. Figure 5 presents the structural model results.

## 4. Discussion

The CC-PACE model integrates the 5A’s framework of creativity with the I-PACE model of Internet-use disorders to explain students’ AI dependence during the creative process. The present study provides empirical support for most of the proposed relationships, with 12 of the 13 hypotheses supported. Specifically, CPI and CSE were found to significantly and positively associated with CA and PAICA, and both CA and PAICA significantly and positively associated with students’ TS, UP, and PDA, which reflect different levels of AI reliance during AI-assisted creation. In addition, TS and UP further related to CSA, whereas PDA did not. The following sections discuss these findings in turn.

### 4.1. Personal Characteristics and Affective/Cognitive Responses

The results showed that students’ personal characteristics were associated with their affective and cognitive responses during the C-creation processes. With regard to affective responses, students with stronger creative self-concepts tended to report greater psychological closeness to the audience. One possible explanation is that these students may regard creative activities as an important part of their creative identity. This may lead them to perceive audience interactions as more self-relevant and to develop a stronger sense of psychological closeness to the audience. Similarly, students with stronger creative self-concepts may engage in creative interactions with greater confidence and more positive expectations about audience responses, which may make them more likely to anticipate or interpret such responses as supportive (Rennick, 2021; Romney et al., 2024). Such perceived support may contribute to a stronger sense of psychological closeness to the audience (Bagci et al., 2023; Weinstein et al., 2019).

With regard to cognitive responses, students with stronger creative self-concepts tended to report higher recognition of AI’s creator role. These students may be more sensitive to creative cues and processes, and thus more likely to acknowledge the utility of AI in such C-creative tasks. This finding is consistent with research on technology acceptance modeling (TAM) (K. Li, 2025; Paredes-Aguirre et al., 2026; Zhao et al., 2025). Relatedly, H. Li et al. (2025) found that students’ perceptions of AI’s functions also shape their CPI and CSE. Together with the present findings, this suggests a potentially reciprocal relationship between students’ creative self-concept and their perceptions of AI’s role as a creator.

Overall, the findings provide empirical support for the CC-PACE model’s proposition that students’ personal characteristics are associated with their affective and cognitive responses in C-creation processes.

These findings also provide preliminary validity evidence for the adapted PAICA scale, suggesting that its items could capture students’ perceptions of AI as a creator. The adapted scale may serve as a useful instrument for assessing such perceptions.

### 4.2. Affective/Cognitive Responses and AI Dependence

The results showed that students’ affective and cognitive responses were associated with their AI dependence during the C-creation processes. With regard to affective responses, students with greater psychological closeness to the audience tended to report greater AI dependence. One possible explanation is that students who feel psychologically closer to their audience may perceive that audience as more accepting of their creative practices, including their use of AI. Such perceived acceptance may reduce concerns about the acceptability of AI use, thereby contributing to greater AI dependence. Another possible explanation is that audience closeness may increase students’ awareness of audience expectations and strengthen their motivation to produce high-quality creative work. When students perceive the audience as supportive or important, they may experience a stronger sense of responsibility toward the creative outcome (Hu et al., 2023). In this context, AI may be adopted as a strategic resource to help students meet perceived audience expectations and improve the quality of their creative outputs (Doshi & Hauser, 2024; Kim & Lee, 2022).

Similarly, students with stronger perceptions of AI’s role as a creator also tended to report greater AI dependence. This association may be because when students perceive that AI can contribute to their creative activities, they are more likely to use this tool. This interpretation is consistent with findings from TAM studies, which show that perceived usefulness is a key predictor of technology use (Hu & Gong, 2025; Kelly et al., 2023; Zou et al., 2023).

Overall, the findings provide empirical support for the CC-PACE model’s proposition that students’ affective and cognitive responses are associated with their AI dependence during the C-creation processes.

These findings also provide measurement-related evidence for the adapted addiction scale used in this study. The three dimensions, namely TS, UP, and PDA, captured distinct aspects of AI dependence during C-creation. This suggests that the adapted scale may support a more differentiated assessment of AI dependence during C-creation processes.

### 4.3. AI Dependence and Creative Product Evaluation

The results indicated that the TS and UP dimensions of students’ AI dependence were associated with self-assessments of their C-creation products. Higher scores on these dimensions indicated greater AI dependence, reflected in more time spent using AI and stronger willingness to use AI. Such AI use may influence two aspects of students’ self-assessments. Regarding the actual quality of the work, AI assistance may improve its creativity. Recent studies have shown that products created with AI were more creative than works created without AI assistance (Doshi & Hauser, 2024; Lee & Chung, 2024; H. Wang et al., 2026). In terms of students’ self-judgment, AI assistance may lead them to overestimate the creativity of their own work. Self-perception theory suggests that individuals form self-evaluations by observing their own behavior (Dico, 2018). When students believe that AI can enhance their creative work and actively apply it, they may develop more positive self-evaluations based on this behavior (Urban et al., 2024).

However, the PDA dimension was not found to be significantly associated with students’ self-assessments. This finding suggests that students who experience greater difficulty creating without AI do not necessarily evaluate their creative outcomes more positively after AI-assisted creation. On the one hand, the quality of these students’ work may not have actually improved. Higher PDA scores indicate greater perceived difficulty in creating independently. These students may use AI primarily to compensate for barriers or limitations in practical skills, rather than to enhance the quality of their work. Under such circumstances, the quality of their outputs may not actually improve. On the other hand, these students may underestimate the quality of their own work. Unlike TS and UP, PDA could reflect the relative contribution of humans and AI. Higher PDA indicates lower independence in C-creation and greater difficulty completing the work through one’s own effort. Drawing on Smith and Newman’s (2014) research on collective creation, students’ evaluations of work quality depend not only on the final work itself but also on how much they believe they contributed to it. For students with high PDA, AI might be perceived as having contributed more to the final work, whereas students’ own contribution may be viewed as relatively limited. This perceived shift in contribution further shapes students’ self-evaluation of creative outcomes. Thus, even if AI involvement improves the quality of the work, students with high PDA may not necessarily report higher CSA.

Most of the results of hypothesis testing in this study were consistent with previous findings, suggesting that, in the context of C-creativity, personal characteristics, affective and cognitive responses, AI dependence, and creative product evaluations of creative works are interrelated. However, building on prior research, this study revealed several points that warrant attention. First, prior research has emphasized that students should understand the functions of AI tools in order to use them more effectively and has conceptualized knowledge of the functions and applications of AI tools as a component of AI literacy (H. Li et al., 2025). This study acknowledges the importance of such tool understanding. However, the findings suggest that greater familiarity with AI functions and applications may also be associated with greater dependence on AI. Therefore, AI literacy education should place greater emphasis on self-monitoring, helping students avoid over-delegating creative judgment to AI and thereby reducing the risk of developing AI dependence. Second, prior studies have suggested that AI tools can enhance creative performance or improve evaluations of creative works by supporting idea generation, reducing cognitive load, and providing immediate feedback (Kreijkes et al., 2026; Urban et al., 2024). These studies highlight the supportive and empowering role of AI in the C-creation processes. In contrast to these relatively positive findings, this study shows that the relationship between AI use and the evaluation of creative works is not consistently positive. In particular, PDA was not associated with CSA. The findings further suggest that when AI becomes an object of dependence from which students find it difficult to disengage, it may not necessarily strengthen their positive perceptions. Therefore, teachers should guide students to think independently before using AI-based assistive tools during C-creation (Wong & Qiu, 2026). In doing so, students may be better able to maintain creative agency and independence in the age of AI.

### 4.4. Contributions

#### 4.4.1. Theoretical Contributions

This study makes several contributions to the field. The first contribution is the development of the CC-PACE model, which offers an integrative analytical framework for examining AI dependence in the context of C-creation. As AI support becomes increasingly prevalent, understanding AI dependence in C-creation has attracted growing scholarly attention. In the “Decalogue” of research challenges in creativity research, proposed by 30 scholars (Corazza et al., 2025), AI dependence in C-creation is identified as a central issue cutting across multiple research dimensions. The CC-PACE model addresses this challenge by providing an integrated theoretical lens for systematic investigation.

Second, this study advances existing theoretical models by integrating and extending both the 5A’s framework of creativity and the I-PACE model. The 5A’s framework emphasizes that its elements are interrelated and indivisible, yet it does not explicate the systematic mechanisms linking them (Glăveanu, 2013). Building on this foundation, this study elaborates on the relationships among the 5A’s elements and updates the framework by incorporating AI creator roles and C-creative processes. At the same time, this study extends the applicability of the I-PACE model to the domain of C-creation, and further validates the roles of students’ personal characteristics, as well as their affective and cognitive responses, in shaping AI dependence and its consequences.

#### 4.4.2. Practical Implications

From a practical perspective, the primary contribution of this study is to demonstrate that students’ affective and cognitive responses were significantly associated with AI dependency in C-creation. This finding provides a practical basis for educators seeking to prevent and address students’ AI dependence. Based on the findings, three priority interventions are proposed.

First, the role of AI in C-creative processes should be appropriately positioned. Teachers should guide students to view AI as a tool rather than as an independent or dominant creative agent. By clearly distinguishing between the supportive functions of AI and students’ own creative responsibilities, teachers could discourage students from assigning excessive creative agency to AI and help students recognize that they remain the central agents of creative ideation, judgment, and decision-making.

Second, students’ agency in C-creation should be strengthened. Teachers should encourage students to think independently before using AI tools and provide training in self-monitoring and critical thinking. Students should also learn to consciously regulate the degree of AI involvement according to the specific requirements of different stages of C-creation. By emphasizing the centrality of human creative judgment in C-creative processes (McGuire et al., 2024; OECD, 2026), teachers could help students keep their use of AI within safe and pedagogically appropriate boundaries. This approach may reduce the potential negative consequences of AI overdependence.

Third, greater emphasis should be placed on process-oriented assessment in C-creation. An exclusive focus on final products may lead students to overlook their own participation in the creative process and may allow AI use to shift from purposeful support to habitual or even dependent behavior. Therefore, teachers should assess not only final creative products but also students’ involvement in ideation, decision-making, revision, and reflection. Process-oriented assessment could encourage students to focus on the development of their own creative abilities.

### 4.5. Limitations and Future Research

Despite the important contributions of this study, several limitations remain to be addressed and refined in future research.

**Figure 6:**
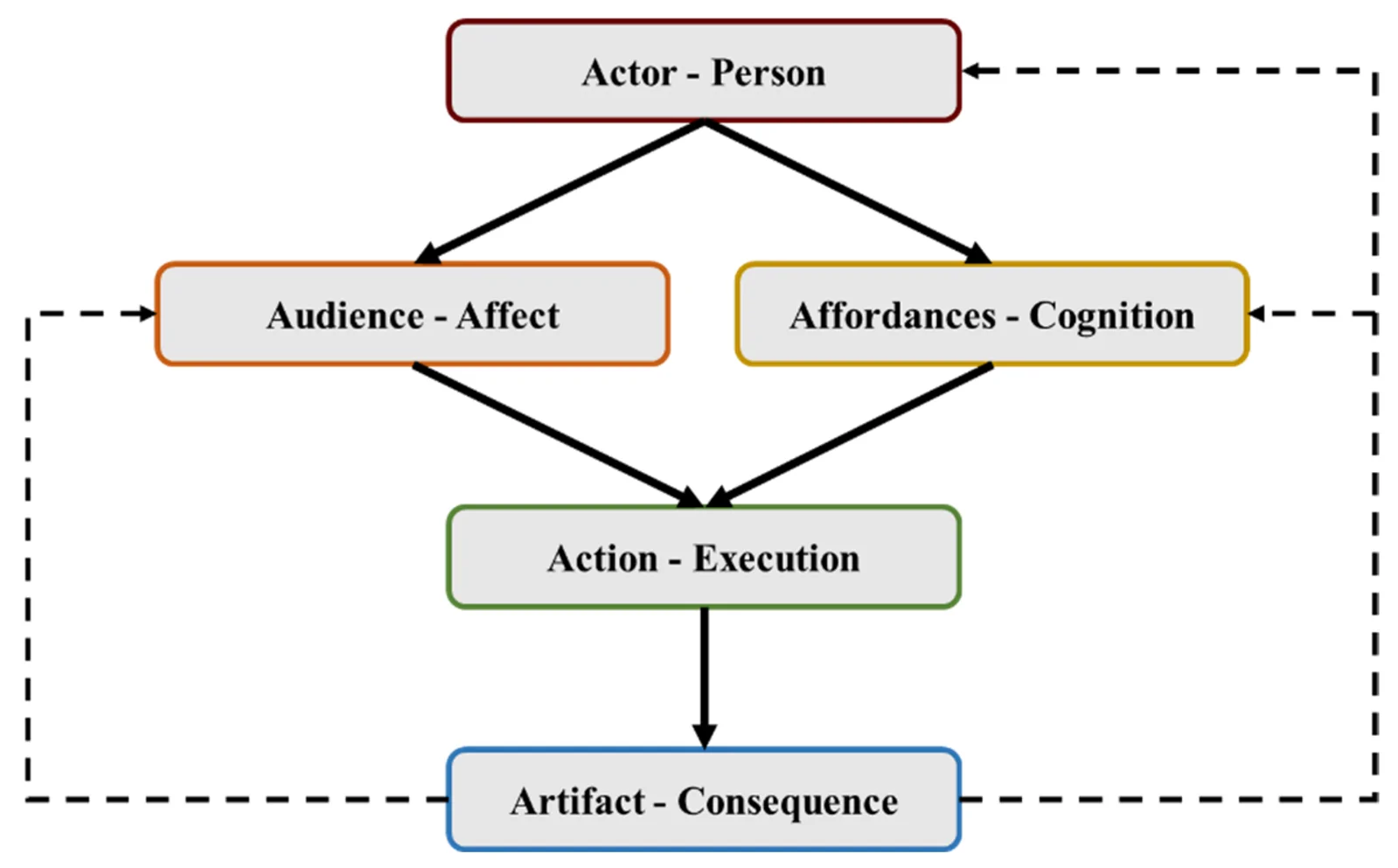
Cyclical interaction pathways in the CC-PACE model.

First, CC-PACE is a preliminary integrative model that combines the 5A’s framework and the I-PACE model, and several conceptual and empirical gaps warrant further investigation. (1) Additional constructs related to C-creation could be incorporated into each component of the CC-PACE model. For example, creative personality factors could be further included in the Actor-Person component; audience pressure could be considered within the Audience-Affect component; and Artifact-Consequence could be measured not only in terms of behavioral or learning outcomes, but also from the perspective of others’ evaluations of creative artifacts. (2) The relationship between the Audience-Affect component and the Affordances-Cognition component within C-creation remains underexplored and warrants further empirical examination. For instance, future studies could examine whether perceived audience pressure is associated with the extent to which students regard AI as a creative agent. (3) Only the core structural pathways of CC-PACE (solid line in Figure 6) were empirically validated in this study, whereas possible reciprocal or feedback effects were not examined. Drawing on the I-PACE model, the Artifact-Consequence may exert a reinforcing or feedback effect on other components of the model (dotted line in Figure 6) (Brand et al., 2016, 2025). For example, the consequences of AI use may further influence students’ audience-related emotional experiences, views on AI, and creative self-efficacy. Whether such reciprocal pathways or cyclical action processes emerge in C-creation contexts remains to be empirically validated.

Second, this study was conducted among college students in China. Although this context provides valuable insights into AI dependence in Chinese higher education, the generalizability of the findings to other cultural or national contexts may be limited. Future studies should conduct cross-cultural replications with samples from different countries and educational systems to broaden the understanding of the antecedents and consequences of AI dependency.

Third, no formal pilot study or preliminary testing was conducted before the modified questionnaire was administered. Although the adapted measures demonstrated acceptable reliability and construct validity in the main study, the absence of preliminary testing may have limited a comprehensive assessment of the modified items. Future research should further validate these aspects of the modified items in relevant research contexts.

Finally, all data in this study were collected through students’ self-reports, which may introduce response bias and raise concerns about common method variance, as the use of a single data source may inflate the observed relationships among variables. In addition, the cross-sectional design limits our ability to infer the temporal ordering and causal relationships among the five constructs of the CC-PACE model. Future research could address these limitations through multi-method or mixed-method designs that combine self-report measures with qualitative interviews, learning analytics, or behavioral indicators to reduce reliance on a single data source. Longitudinal designs are also needed to examine changes in AI dependence and clarify the temporal ordering among the five constructs. In addition, experimental studies that manipulate the level or type of AI involvement could provide stronger evidence for the causal pathways proposed in the CC-PACE model.

### 4.6. Conclusions

This study developed the CC-PACE model by integrating the 5A’s framework and the I-PACE model, and explored students’ AI dependency in C-creation contexts. The results are as follows: (1) CPI and CSE significantly and positively predict CA and PAICA; (2) CA and PAICA significantly and positively predict AI dependency in C-creation; and (3) TS and UP in students’ AI use in C-creation significantly and positively predict CSA, whereas PDA is not significantly associated with CSA. Overall, the findings provide empirical support for the CC-PACE model.

These findings extend existing research on AI-assisted creativity by providing empirical support for the CC-PACE model. It offers an integrative framework for understanding students’ dependence on AI in C-creation contexts. The findings suggest that this dependence is not merely a technical-use issue, but is associated with personal creative factors, affective experiences, cognitive perceptions of AI, and the perceived consequences of AI-supported creative work.

The study also offers pedagogical implications for educational practice, suggesting that teachers should guide students to appropriately position the role of AI in the C-creation process, strengthen students’ agency, and place greater emphasis on evaluating the creation process.

By identifying and addressing the factors associated with AI dependence in C-creation, educators and researchers can better support the balanced and sustainable development of adolescents’ creativity.

## Figures and Tables

**Table 1:** Factors examined in this study.

Associated 5A Elements	Associated PACE Components	Factors		Abbreviations
		Measured Factors	Measured Subfactors	
Actor	Person	Self-Concept of Creativity	Creative Personal Identity	CPI
			Creative Self-Efficacy	CSE
Audience	Affect	Closeness with the Audience	Closeness with the Audience	CA
Affordances	Cognition	AI Roles	Perception of AI as a Creative Agent	PAICA
Action	Execution	AI Dependence in C-creation	Time Spent	TS
			Usage Preferences	UP
			Perceived Difficulty in Avoiding	PDA
Artifact	Consequence	Creative Assessment	Creative Self-Assessment	CSA

**Table 2:** Demographic information of the participants.

		Frequency	Percent
Gender	Male	513	63.10%
	Female	300	36.90%
Grade	Freshman year	569	69.99%
	Sophomore year	222	27.31%
	Junior year	20	2.46%
	Senior year	2	0.25%

**Table 3:** The adapted PAICA scale based on Siemon’s (2022) framework.

Original Item	Adapted Item
- Is good at finding many and new possible solutions for situations.	- I think AI is good at finding many and new possible solutions for situations.
- Conducts research, to develop something new based on it.	- I think AI could conduct research, to develop something new based on it.
- Is always looking for new ideas and developments.	- I think AI is always looking for new ideas and developments.
- Is good at uncovering novel patterns and form new associations.	- I think AI is good at uncovering novel patterns and forming new associations.
- Is good at innovative problem-solving.	- I think AI is good at innovative problem-solving.
- Is good at contributing expert knowledge to a complex task	- I think AI is good at contributing expert knowledge to a complex task

**Table 4:** The adapted addiction scale based on Andreassen et al. (2012).

Variables	Original Item	Adapted Item
TS	- Spent a lot of time thinking about…	- Spent a lot of time asking AI to determine the creative direction.
		- Spent a lot of time asking AI to generate creative inspiration or ideas.
		- Spent a lot of time asking AI to select the best idea from multiple ideas.
		- Spent a lot of time asking AI to generate the work based on a particular idea.
UP	- Felt an urge to use… more and more?	- Felt an urge to use AI to determine the creative direction more and more?
		- Felt an urge to use AI to generate creative inspiration or ideas more and more?
		- Felt an urge to use AI to select the best idea from multiple ideas more and more?
		- Felt an urge to use AI to turn a particular idea into an actual work more and more?
PDA	- Tried to cut down on the use of… without success?	- Tried to cut down on the use of AI to help me determine the creative direction without success?
		- Tried to cut down on the use of AI to help me generate creative inspiration or ideas without success?
		- Tried to cut down on the use of AI to help me select the best idea from multiple ideas without success?
		- Tried to cut down on the use of AI to help me turn ideas into actual works without success?

**Table 5:** Descriptive statistics.

	Min	Max	Mean	SD
CA	1.00	5.00	2.263	0.772
CPI	1.00	4.00	1.865	0.660
CSA	1.00	5.00	2.275	0.703
CSE	1.00	5.00	2.163	0.733
PAICA	1.00	5.00	2.009	0.709
PDA	1.00	5.00	2.603	0.842
TS	1.00	5.00	2.241	0.763
UP	1.00	5.00	2.347	0.770

**Table 6:** Reliability and convergent validity.

	Cronbach’s Alpha	Composite Reliability	AVE
CA	0.908	0.942	0.845
CPI	0.913	0.935	0.741
CSA	0.888	0.93	0.817
CSE	0.941	0.953	0.772
PAICA	0.933	0.947	0.749
PDA	0.94	0.957	0.848
TS	0.91	0.937	0.788
UP	0.914	0.939	0.795

**Table 7:** Fornell-Larcker criterion.

	CA	CPI	CSA	CSE	PAICA	PDA	TS	UP
CA	0.919							
CPI	0.444	0.861						
CSA	0.737	0.443	0.904					
CSE	0.502	0.750	0.519	0.879				
PAICA	0.500	0.537	0.533	0.579	0.866			
PDA	0.389	0.235	0.446	0.311	0.356	0.921		
TS	0.520	0.419	0.602	0.491	0.540	0.521	0.888	
UP	0.494	0.341	0.584	0.395	0.508	0.687	0.743	0.892

**Table 8:** HTMT.

	CA	CPI	CSA	CSE	PAICA	PDA	TS
CPI	0.486						
CSA	0.822	0.491					
CSE	0.542	0.812	0.567				
PAICA	0.542	0.579	0.585	0.615			
PDA	0.419	0.254	0.486	0.329	0.378		
TS	0.571	0.459	0.669	0.529	0.584	0.563	
UP	0.541	0.372	0.645	0.425	0.547	0.741	0.815

**Table 9:** Structural model results and hypothesis testing results.

Hypothesis	Path	*β*	SE	*t*-Value	*p*-Value	f^2^	95% Bootstrap CI		Supported
							2.50%	97.50%	
H1	CPI → CA	0.153	0.054	2.845	0.004	0.014	0.049	0.260	Yes
H2	CPI → PAICA	0.234	0.060	3.925	0.000	0.037	0.123	0.355	Yes
H3	CSE → CA	0.387	0.055	7.084	0.000	0.089	0.279	0.490	Yes
H4	CSE → PAICA	0.403	0.060	6.761	0.000	0.111	0.285	0.514	Yes
H5	CA → TS	0.333	0.043	7.687	0.000	0.133	0.248	0.419	Yes
H6	CA → UP	0.320	0.046	7.004	0.000	0.116	0.230	0.408	Yes
H7	CA → PDA	0.281	0.048	5.841	0.000	0.073	0.132	0.375	Yes
H8	PAICA → TS	0.374	0.042	8.867	0.000	0.168	0.291	0.456	Yes
H9	PAICA → UP	0.347	0.044	7.894	0.000	0.136	0.262	0.434	Yes
H10	PAICA → PDA	0.216	0.042	5.101	0.000	0.043	0.187	0.300	Yes
H11	TS → CSA	0.374	0.051	7.324	0.000	0.106	0.274	0.471	Yes
H12	UP → CSA	0.251	0.058	4.324	0.000	0.035	0.136	0.361	Yes
H13	PDA → CSA	0.079	0.041	1.930	0.054	0.006	0.002	0.162	Not

**Table 10:** R-square (R^2^) values and predictive relevance (Q^2^).

Outcome Variable	R^2^	Q^2^
CA	0.262	0.256
CSA	0.407	0.204
PAICA	0.359	0.353
PDA	0.186	0.088
TS	0.375	0.231
UP	0.335	0.155

## Data Availability

The data presented in this study are available on request from the corresponding author due to privacy.

## Abbreviations

The following abbreviations are used in this manuscript:

AI	Artificial Intelligence
ISSCI	International Society for the Study of Creativity and Innovation
I-PACE	Interaction of Person–Affect–Cognition–Execution
CC-PACE	C-Creativity of Person–Affect–Cognition–Execution
CPI	Creative Personal Identity
CSE	Creative Self-Efficacy
CA	Closeness with the Audience
PAICA	Perception of AI as a Creative Agent
TS	Time Spent
UPs	Usage Preferences
PDA	Perceived Difficulty in Avoiding
CSA	Creative Self-Assessment
PLS-SEM	Partial Least Squares Structural Equation Modeling
CR	Composite Reliability
AVE	Average Variance Extracted
HTMT	Heterotrait–Monotrait Ratio of Correlations
VIF	Variance Inflation Factor

## References

[B1-jintelligence-14-00155] Amabile T. M. (1982). Social psychology of creativity: A consensual assessment technique. Journal of Personality and Social Psychology.

[B2-jintelligence-14-00155] Andreassen C. S., Torsheim T., Brunborg G. S., Pallesen S. (2012). Development of a Facebook addiction scale. Psychological Reports.

[B3-jintelligence-14-00155] Bagci S., Turnuklu A., Tercan M., Cameron L., Turner R. (2023). Have some confidence in contact: Self-efficacy beliefs among children moderate the associations between cross-group friendships and outgroup attitudes. Journal of Applied Social Psychology.

[B4-jintelligence-14-00155] Bai Y. Q. (2026). An experimental study on the impact of generative AI on university students’ emotions and performance in creative problem-solving tasks. Learning and Instruction.

[B5-jintelligence-14-00155] Bakker M. A., Chadwick M. J., Sheahan H. R., Tessler M. H., Campbell-Gillingham L., Balaguer J., McAleese N., Glaese A., Aslanides J., Botvinick M. M., Summerfield C. (2022). Fine-tuning language models to find agreement among humans with diverse preferences. 36th international conference on neural information processing systems.

[B6-jintelligence-14-00155] Barón-Birchenall L., Sánchez-Vallejo A., Toro-Silva C., Folleco-Eraso A. (2025). Creativity: An individual or collective phenomenon? A historical-psychological perspective. Adaptive Behavior.

[B7-jintelligence-14-00155] Baudier P., De Vassoigne T., Arami M., Delannoy A., Germon R. (2025). Embracing the metaverse: User perception and acceptance of the metaverse in education. Computers in Human Behavior.

[B8-jintelligence-14-00155] Berretta S., Tausch A., Ontrup G., Gilles B., Peifer C., Kluge A. (2023). Defining human-AI teaming the human-centered way: A scoping review and network analysis. Frontiers in Artificial Intelligence.

[B9-jintelligence-14-00155] Brand M., Müller A., Wegmann E., Antons S., Brandtner A., Müller S. M., Stark R., Steins-Loeber S., Potenza M. N. (2025). Current interpretations of the I-PACE model of behavioral addictions. Journal of Behavioral Addictions.

[B10-jintelligence-14-00155] Brand M., Young K. S., Laier C. (2014). Prefrontal control and internet addiction: A theoretical model and review of neuropsychological and neuroimaging findings. Frontiers in Human Neuroscience.

[B11-jintelligence-14-00155] Brand M., Young K. S., Laier C., Wölfling K., Potenza M. N. (2016). Integrating psychological and neurobiological considerations regarding the development and maintenance of specific Internet-use disorders: An Interaction of Person-Affect-Cognition-Execution (I-PACE) model. Neuroscience & Biobehavioral Reviews.

[B12-jintelligence-14-00155] Cao Y. J., Wang F. (2025). Generative AI streamers in action: A source credibility perspective. International Journal of Advertising.

[B13-jintelligence-14-00155] Casale S., Caplan S. E., Fioravanti G. (2016). Positive metacognitions about Internet use: The mediating role in the relationship between emotional dysregulation and problematic use. Addictive Behaviors.

[B14-jintelligence-14-00155] Corazza G. E., Agnoli S., Jorge Artigau A., Beghetto R. A., Bonnardel N., Coletto I., Faiella A., Gerardini K., Gilhooly K., Glăveanu V. P., Hanson M. H., Kapoor H., Kaufman J. C., Kenett Y. N., Kharkhurin A. V., Luchini S., Mangion M., Mirabile M., Obialo F., Lubart T. (2025). Cyber-creativity: A decalogue of research challenges. Journal of Intelligence.

[B15-jintelligence-14-00155] Deryakulu D., Ursavaş Ö. F. (2014). Genetic and environmental influences on problematic Internet use: A twin study. Computers in Human Behavior.

[B16-jintelligence-14-00155] Dico G. L. (2018). Self-perception theory, radical behaviourism, and the publicity/privacy issue. Review of Philosophy and Psychology.

[B17-jintelligence-14-00155] Dong G., Potenza M. N. (2014). A cognitive-behavioral model of Internet gaming disorder: Theoretical underpinnings and clinical implications. Journal of Psychiatric Research.

[B18-jintelligence-14-00155] Dong G., Zhou H., Zhao X. (2011). Male Internet addicts show impaired executive control ability: Evidence from a color-word Stroop task. Neuroscience Letters.

[B19-jintelligence-14-00155] Doshi A. R., Hauser O. P. (2024). Generative AI enhances individual creativity but reduces the collective diversity of novel content. Science Advances.

[B20-jintelligence-14-00155] Ebeling-Witte S., Frank M. L., Lester D. (2007). Shyness, internet use, and personality. CyberPsychology & Behavior.

[B21-jintelligence-14-00155] Faul F., Erdfelder E., Buchner A., Lang A. (2009). Statistical power analyses using G*Power 3.1: Tests for correlation and regression analyses. Behavior Research Methods.

[B22-jintelligence-14-00155] Faul F., Erdfelder E., Lang A., Buchner A. (2007). G*Power 3: A flexible statistical power analysis program for the social, behavioral, and biomedical sciences. Behavior Research Methods.

[B23-jintelligence-14-00155] Glăveanu V. P. (2013). Rewriting the language of creativity: The five A’s framework. Review of General Psychology.

[B24-jintelligence-14-00155] Goldstein R. Z., Volkow N. D. (2011). Dysfunction of the prefrontal cortex in addiction: Neuroimaging findings and clinical implications. Nature Reviews Neuroscience.

[B25-jintelligence-14-00155] Grossen M. (2008). Methods for studying collaborative creativity: An original and adventurous blend. Thinking Skills and Creativity.

[B26-jintelligence-14-00155] Hair J. F., Sarstedt M., Ringle C. M. (2019). Rethinking some of the rethinking of partial least squares. European Journal of Marketing.

[B27-jintelligence-14-00155] Hsieh Y., Shen A. C., Wei H., Feng J., Huang S. C., Hwa H. (2016). Associations between child maltreatment, PTSD, and internet addiction among Taiwanese students. Computers in Human Behavior.

[B28-jintelligence-14-00155] Hu X., Gong W. (2025). Modeling Chinese EFL learners’ intention to use generative AI for L2 writing through an integrated model of the TAM and TTF. Education and Information Technologies.

[B29-jintelligence-14-00155] Hu X., Zhang J. Q., Shen S. (2023). Exploring the pathway from seeking to sharing social support in e-learning: An investigation based on the norm of reciprocity and expectation confirmation theory. Current Psychology.

[B30-jintelligence-14-00155] Jiang Y. P., Zhao B. H., Qin X. Y., Wu X. (2025). Network analysis of psychosocial correlates of short video addiction in Chinese adolescents: A study guided by the I-PACE model. Current Psychology.

[B31-jintelligence-14-00155] Karwowski M., Lebuda I., Wisniewska E., Gralewski J. (2013). Big five personality traits as the predictors of creative self-efficacy and creative personal identity: Does gender matter?. The Journal of Creative Behavior.

[B32-jintelligence-14-00155] Kelly S., Kaye S., Oviedo-Trespalacios O. (2023). What factors contribute to the acceptance of artificial intelligence? A systematic review. Telematics and Informatics.

[B33-jintelligence-14-00155] Kim J., Lee S. (2022). Are two heads better than one? The effect of student-AI collaboration on students’ learning task performance. TechTrends.

[B34-jintelligence-14-00155] Kreijkes P., Kewenig V., Kuvalja M., Lee M., Hofman J. M., Vitello S., Sellen A., Rintel S., Goldstein D. G., Rothschild D., Tankelevitch L., Oates T. (2026). Effects of LLM use and note-taking on reading comprehension and memory: A randomised experiment in secondary schools. Computers & Education.

[B35-jintelligence-14-00155] Lee B. C., Chung J. J. (2024). An empirical investigation of the impact of ChatGPT on creativity. Nature Human Behaviour.

[B36-jintelligence-14-00155] Li H., Zhang Y., Chen M., Zhao T., Jou M. (2025). Creative personal identity in the age of generative AI: A social-cognitive pathway of AI literacy, self-efficacy, and mindset. Computers in Human Behavior.

[B37-jintelligence-14-00155] Li K. (2025). Factors influencing learners learning interest when using AI chatbots-assisted math leaning in higher education. Frontiers in Psychology.

[B38-jintelligence-14-00155] Magno F., Cassia F., Ringle C. M. (2022). A brief review of partial least squares structural equation modeling (PLS-SEM) use in quality management studies. The TQM Journal.

[B39-jintelligence-14-00155] McGuire J., De Cremer D., Van de Cruys T. (2024). Establishing the importance of co-creation and self-efficacy in creative collaboration with artificial intelligence. Scientific Reports.

[B40-jintelligence-14-00155] OECD (2026). OECD digital education outlook 2026: Exploring effective uses of generative AI in education.

[B41-jintelligence-14-00155] Paredes-Aguirre M., Bolanos-Mendoza C., Ortiz-Rojas M., Martinez-Tovar J., Sanchez-Mendoza M., Moreno-Abramowicz M. (2026). Understanding perceived learning and the role of digital self-efficacy in generative AI adoption among STEM and non-STEM students. Cogent Education.

[B42-jintelligence-14-00155] Rafner J., Zana B., Dalsgaard P., Biskjaer M. M., Sherson J. (2023). Picture this: AI-assisted image generation as a resource for problem construction in creative problem-solving. 15th conference on creativity and cognition.

[B43-jintelligence-14-00155] Rennick S. (2021). Self-fulfilling prophecies. Philosophies.

[B44-jintelligence-14-00155] Rhodes M. (1961). An analysis of creativity. The Phi Delta Kappan.

[B45-jintelligence-14-00155] Romney A., Harrison J. T., Benson S. (2024). Looking back to predict the future: A review of empirical support for the self-fulfilling prophecy. Management Research Review.

[B46-jintelligence-14-00155] Sabrina H. (2026). AI-mediated creative agency: A framework for creative self-efficacy in human-AI collaboration.

[B47-jintelligence-14-00155] Sidra S., Mason C. (2026). Generative AI in human-AI collaboration: Validation of the collaborative AI literacy and collaborative AI metacognition scales for effective use. International Journal of Human–Computer Interaction.

[B48-jintelligence-14-00155] Siemon D. (2022). Elaborating team roles for artificial intelligence-based teammates in human-AI collaboration. Group Decision and Negotiation.

[B49-jintelligence-14-00155] Smith R. K., Newman G. E. (2014). When multiple creators are worse than one: The bias toward single authors in the evaluation of art. Psychology of Aesthetics, Creativity, and the Arts.

[B50-jintelligence-14-00155] Stanciu D., Calugar A. (2022). What is irrational in fearing to miss out on being online. An application of the I-PACE model regarding the role of maladaptive cognitions in problematic internet use. Computers in Human Behavior.

[B51-jintelligence-14-00155] Starcke K., Brand M. (2012). Decision making under stress: A selective review. Neuroscience & Biobehavioral Reviews.

[B52-jintelligence-14-00155] Sun X. H., Qin J. X. (2024). AI for supporting the freedom of drawing. Machine Intelligence Research.

[B53-jintelligence-14-00155] Thornhill-Miller B., Camarda A., Mercier M., Burkhardt J., Morisseau T., Bourgeois-Bougrine S., Vinchon F., El Hayek S., Augereau-Landais M., Mourey F., Feybesse C., Sundquist D., Lubart T. (2023). Creativity, critical thinking, communication, and collaboration: Assessment, certification, and promotion of 21st century skills for the future of work and education. Journal of Intelligence.

[B54-jintelligence-14-00155] Urban M., Děchtěrenko F., Lukavský J., Hrabalová V., Svacha F., Brom C., Urban K. (2024). ChatGPT improves creative problem-solving performance in university students: An experimental study. Computers & Education.

[B55-jintelligence-14-00155] Wang C. W., Ho R. T. H., Chan C. L. W., Tse S. (2015). Exploring personality characteristics of Chinese adolescents with internet-related addictive behaviors: Trait differences for gaming addiction and social networking addiction. Addictive Behaviors.

[B56-jintelligence-14-00155] Wang H., Da T., Wang Y., Luo F., Zhang Y., Tlili A., Yang D., Wang Y., Xu J., Zhu X., Wan M., Huang R. (2026). Augmentation or limitation? Generative AI’s influence on college students’ creative problem-solving. Thinking Skills and Creativity.

[B57-jintelligence-14-00155] Wang X. M., Wu Z. L., Huang W. Q., Wei Y. T., Huang Z. S., Xu M. L., Chen W. (2023). VIS plus AI: Integrating visualization with artificial intelligence for efficient data analysis. Frontiers of Computer Science.

[B58-jintelligence-14-00155] Wegmann E., Stodt B., Brand M. (2015). Addictive use of social networking sites can be explained by the interaction of Internet use expectancies, Internet literacy, and psychopathological symptoms. Journal of Behavioral Addictions.

[B59-jintelligence-14-00155] Weinstein N., Vansteenkiste M., Paulmann S. (2019). Listen to your mother: Motivating tones of voice predict adolescents’ reactions to mothers. Developmental Psychology.

[B60-jintelligence-14-00155] Wong S. S. H., Qiu S. X. (2026). Think first, ChatGPT later: Guiding human–AI collaboration for learning gains in independent human creativity. Educational Psychology Review.

[B61-jintelligence-14-00155] Ye J., Zhang M., Nong W., Wang L., Yang X. (2025). The relationship between inert thinking and ChatGPT dependence: An I-PACE model perspective. Education and Information Technologies.

[B62-jintelligence-14-00155] Young K. S., Brand M. (2017). Merging theoretical models and therapy approaches in the context of internet gaming disorder: A personal perspective. Frontiers in Psychology.

[B63-jintelligence-14-00155] Zhang S., Zhao X., Zhou T., Kim J. H. (2024). Do you have AI dependency? The roles of academic self-efficacy, academic stress, and performance expectations on problematic AI usage behavior. International Journal of Educational Technology in Higher Education.

[B64-jintelligence-14-00155] Zhang Y., Cheng Z., Pan Y., Xu Y. (2022). Psychological antecedents and consequences of social integration based on self-disclosure in virtual communities: Empirical evidence from Sina Microblog. Frontiers in Psychology.

[B65-jintelligence-14-00155] Zhao Z. X., An Q., Liu J. Q. (2025). Exploring AI tool adoption in higher education: Evidence from a PLS-SEM model integrating multimodal literacy, self-efficacy, and university support. Frontiers in Psychology.

[B66-jintelligence-14-00155] Zier J., Diakopoulos N. (2026). Beyond the byline: Audience expectations for AI disclosure in news media. Digital Journalism.

[B67-jintelligence-14-00155] Zou B., Lyu Q., Han Y., Li Z., Zhang W. (2023). Exploring students’ acceptance of an artificial intelligence speech evaluation program for EFL speaking practice: An application of the integrated model of technology acceptance. Computer Assisted Language Learning.

[B68-jintelligence-14-00155] Zyphur M. J., Bonner C. V., Tay L. (2023). Structural equation modeling in organizational research: The state of our science and some proposals for its future. Annual Review of Organizational Psychology and Organizational Behavior.

